# Understanding Clinical Learning Quality Aspects in Mental Health Nursing Practice Course among Students: A Comprehensive Examination

**DOI:** 10.3390/nursrep14020086

**Published:** 2024-05-10

**Authors:** Gizell Green, Sanaa Adawi

**Affiliations:** 1 Nursing Department, School of Health Sciences, Ariel University, Ariel 407000, Israel; 2 Department of Health System Management, School of Health Sciences, Ariel University, Ariel 407000, Israel; sanaa.adawi@mail.huji.ac.il

**Keywords:** clinical learning quality, mental health practice, nursing students, theoretical knowledge, quality of tutorial strategies, learning opportunities, self-directed learning, safety and nursing care quality, learning environment quality

## Abstract

There is a growing demand for comprehensive evaluations of the clinical learning quality of nursing education and the necessity to establish robust predictors and mediators for enhancing its outcomes within the context of mental health practice. This study is threefold: 1. Evaluating nursing students’ clinical learning quality before and after mental health nursing practice; 2. Establish if the grade of a theoretical course in mental health nursing and the student’s perception of their theoretical knowledge level predicts the grade of mental health nursing practice; 3. Explore how model learning opportunities, self-directed learning, safety, and nursing care quality mediate learning environment quality and tutorial strategies quality following mental health nursing practice. Using a before and after the study, 107 undergraduate nursing students at an Israeli university completed a questionnaire and the Clinical Learning Quality Evaluation Index tool to assess their perceptions of clinical learning quality before and after mental health nursing practice. The results showed a decline in students’ perceptions of tutorial strategy quality following mental health practical learning in clinical settings, with the theoretical course grade predicting the practical experience grade and underscoring the mediating role of learning opportunities between the learning environment and tutorial strategies. The study’s findings emphasize the importance of an adaptive learning environment and a solid theoretical foundation in fostering effective tutorial strategies and enhancing the overall learning outcomes for nursing students in mental health education.

## 1. Introduction

Nursing students’ clinical learning quality may significantly influence their learning outcomes. A literature review underlines the multifaceted nature of quality clinical learning, which is impacted by various factors, including the nursing practice environment, clinical learning environment, and nursing students’ perceptions of their clinical learning experiences [1,2,3,4,5,6,7]. Furthermore, the quality of care in mental health units, which is an integral part of nursing education, is linked to the nursing practice environment [8]. This, in turn, has potential implications for nursing students’ future practice. Thus, a comprehensive approach to understanding the state of clinical learning is essential. It includes referencing situational contexts such as the duration and conditions of rotations, the academic settings in which they occur, and the types of mental health care units involved, which can significantly impact student performance.

A multicenter study found that students’ perceptions of their mental health were shaped by factors such as the clinical learning environment and nurse teachers’ clinical learning environment [3]. The quality of the learning environment encompasses satisfaction with the clinical learning experience, the perception of the setting as conducive to learning, and consideration of it as a desirable place for future nursing employment [9]. Furthermore, clinical learning quality can be affected by factors such as lack of staff, lack of learning materials, and overcrowding of patients [10], especially in mental health practical learning in Israel. Therefore, further exploration of these relationships, distinct from the mental health nursing practice course, is essential.

Within the clinical learning quality field, the quality of tutorial strategies holds a particular significance. Tutorial strategy quality involves the preceptor’s guidance in clinical decision making, facilitation of clinical thinking through questioning, support for emotional expression, mediation in challenging patient interactions, enthusiastic teaching of nursing practice, and alignment of final evaluations with feedback [9]. Psychiatry ranked second to last among eight learning areas in a study, highlighting the need for improvements in tutorial strategies within psychiatry education [11,12]. This highlights the need for comprehensive improvements in tutorial strategies within psychiatry education to enhance the clinical learning experience. Several elements may connect to clinical learning quality, which in turn may affect the quality of tutorial strategies. Learning opportunities can be a crucial component of clinical learning quality, anchored in nursing education. Learning opportunities entail being trusted, given autonomy to perform interventions independently, assigned appropriate responsibilities, encouraged to express opinions and critical reflections, respected as a student, and supported during challenging situations [9]. Thus, it is necessary to provide students with experiential learning opportunities, enabling the development of their skills, attitudes, and values [13,14].

Another element is self-directed learning. It can refer to the conscious choice of seeking assistance from others or engaging in independent learning; a study found that readiness for self-directed learning may connect to nursing competency [15]. Therefore, self-directed learning is also a significant component in assessing clinical learning quality.

Safety and nursing care quality represents another pivotal aspect, as nursing students often express their lack of readiness to apply quality and safety skills in real clinical settings [16]. Safety and nursing care quality encompasses nurses adhering to professional standards, ensuring patient safety, providing accessible individual and safety devices, and demonstrating a passion for the nursing profession [9]. Quality learning environments increase students’ perception of a safe clinical environment [17]. Clinical learning quality is also critical in determining the effectiveness of bridging theoretical knowledge and practical skills, particularly in the demanding field of mental health nursing.

Previous studies have explored the relationship between theoretical and clinical performance in nursing education [18,19,20]. A study conducted in the Philippine nursing university found that academic success in theoretical classroom instruction had a positive influence on the clinical or practical phase of nursing education [21]. A qualitative study conducted in Brazil found that nursing students’ perceptions of mental health were influenced by various factors, including academic influences, teachers’ attitudes, and the university environment [22], which is in charge of providing theoretical knowledge. This may suggest that factors such as theoretical knowledge levels, as influenced by academic environments and teachers’ attitudes, can potentially predict the grades attained in practical mental health nursing experience courses. Another study explored the barriers to utilizing theoretical knowledge in clinical settings and found that insufficient theoretical knowledge was a major obstacle to effective performance in clinical settings [23]. Therefore, the literature suggests that theoretical knowledge attainment and practical skill application are closely related in nursing education, particularly in the context of mental health nursing. The application of theoretical knowledge to practical skills may depend upon a favorable and enriching clinical learning quality provided in clinical settings.

Accordingly, the research aims are as follows:

1. Evaluate nursing students’ clinical learning quality regarding tutorial strategies quality, learning opportunities, self-directed learning, safety and nursing care quality, and learning environment quality before and after mental health nursing practice (outcome).
2. Establish if the grade of a theoretical course in mental health nursing and the student’s perception of their mental health theoretical knowledge level predicts the grade of mental health nursing practice (outcome).
3. Explore how model of learning opportunities, self-directed learning, safety and nursing care quality mediate quality of learning environment and tutorial strategies quality following mental health nursing practice (outcome).

The innovation embedded in this study is evident in its holistic assessment of various aspects of clinical learning quality, with a particular focus on the often-ignored dominion of mental health nursing. It incorporates clinical learning quality factors, such as tutorial strategies quality, learning opportunities, self-directed learning, safety and nursing care quality, and learning environment quality, offering a new perspective on clinical learning.

## 2. Materials and Methods

### 2.1. Design

A sample of nursing students provided data through a before and after study design, one-group, two-points-in-time repeated measures study [24].

### 2.2. Setting and Sample

The study was conducted at an Israeli academic institution with a sample of 107 undergraduate nursing students. Inclusion criteria: Participants had to have successfully completed their theoretical clinical course, which is typically taken in a classroom setting at the end of the third year. Exclusion criteria: Participants who have not successfully completed the theoretical clinical course usually take it in a classroom setting at the end of the third year. The participants were selected through convenience sampling. A questionnaire was administered before and after the mental health practical learning unit. The pre-measurement reflects students’ perceptions before starting the mental health practice, while the post-measurement assesses their perceptions upon completing the practice. Consequently, comparing pre- and post-measurements offers valuable insights into the influence of students’ perceptions of tutorial strategy quality and other elements of clinical learning.

A priori sample size estimation was carried out using the G*Power software package (Version 3.1.9.4) to determine the appropriate sample size for the research. This estimation was performed for repeated measures, considering both within and between factors. The following parameters were considered: effect size f = 0.25, α error probability = 0.05, power (1 − β error probability) = 0.80, number of groups = 1, number of measurements = 2, and a correlation of 0.5, repeated measures. This result indicated that a minimum total sample size of N = 52 was needed.

### 2.3. Education in Mental Health Theory and Practice

The mental health nursing curriculum is generic and was developed in partnership with the nursing director, who belongs to the Ministry of Health. It combines two main learning methods: theoretical learning and practical training. Like in other nursing education areas [25]. First, students are required to integrate theoretical and empirical learning within a framework of theoretical mental health courses. Second, the students undergo mental health practical learning and apply the theoretical skills mastered in the theoretical mental health courses in hospital departments. The theoretical learning course lasted for 13 weeks, and was conducted in the fall semester, while the second practical learning spanned 12 shifts, 8 hours per shift during semester of the academic year. Both courses were part of the curriculum for third-year nursing students.

### 2.4. Instruments

The study utilized a three-part questionnaire.

The first part consisted of background information, such as age and gender.

The second part focused on the participants’ reporting perceived learning assessment and grades in the mental health theoretical course and nursing practice. They were asked: “What was your perceived knowledge in mental health nursing level after the theoretical course learning?” and “What grade did you get in the mental health practical training?” Level of knowledge in mental health nursing answer consists of a Likert scale ranging from 1 to 5, where one indicates very low and five indicates very high. Practical training was assessed by clinical instruction using written assessment, with scores ranging from 0 to 100.

The third part included students’ perceptions of the learning environment’s quality using the Clinical Learning Quality Evaluation Index (CLEQI) tool, which is composed of five factors: (a) tutorial strategies quality (six items); (b) learning opportunities (six items); (c) self-directed learning (three items); (d) safety and nursing care quality (four items); and (e) learning environment quality (three items). Each CLEQI factor and the overall CLEQI score ranged from 0 (nothing) to 3 (very much). A higher score reflects students’ perceptions of a higher quality of learning processes. The CLEQI tool had a high internal validity (Cronbach’s alpha of 0.95 overall at the factor level of 0.82 to 0.93) [9,26]. The validity of the Clinical Learning Quality Evaluation Index (CLEQI) tool in mental health nursing experience courses has been affirmed, as indicated by a study examining nursing students’ perceptions of their clinical learning experiences [9].

While several assessment instruments evaluate student nurses’ clinical and educational learning environments [27], it is crucial to acknowledge the distinct nature of the mental health work environment compared to general nursing settings. Consequently, the current study employs the CLEQI tool, which is suitable for the context of mental health nursing.

### 2.5. Ethical Considerations

The study obtained ethical approval from the Institutional Ethics Committee of Ariel University (Approval Code: AU-HEA-GG-20210313; Approval Date: 13 March 2021). Participants provided an electronic informed consent form to an independent researcher, separate from the research team, ensuring independence and minimizing potential biases.

### 2.6. Data Collection and Analysis

The study involved data collection via the completion of a questionnaire at two different points, before (T1) and shortly after (T2) the mental health practical learning experience. Data collection occurred between the beginning of 2021 and the end of 2022. To uphold methodological rigor, a trained research assistant, well-versed in the study’s objectives and procedures, diligently supervised the entirety of the data collection process. Students were accessed via university email invitations with a link to fill out the questionnaire. Out of 450 invited students, 107 responded, resulting in a response rate of 23%. This adherence to standardized data collection methodologies and the significant response rate underscores the reliability and reproducibility of the study’s findings.

The study utilized three software programs for statistical analysis. The first program, G*power, was used to determine the required sample size. The second program was the Statistical Package for the Social Sciences (SPSS TM) version 29.0. Descriptive statistics, such as frequencies, percentages, means, and standard deviations, were employed to sum up demographic variables. A one-way repeated measures analysis of variance was conducted to assess the impact of the mental health nursing practice course at both time points. Correlations were also conducted for T2—following the mental health practical learning experience. The third program, Process software version 4.2, facilitated the execution of a series of regression analyses to examine the path analysis model [28].

## 3. Results

The participants were 107 nursing students from the university with a mean age of 25 (SD = 1.54). The mean exam scores were 92 (SD = 5.63), ranging from 70 to 100; for the frequency and percentages of other background characteristics, see Table 1.

Table 1 presents the participants’ demographic distribution. A minority were male (20; 19%), while the majority were female (87; 81%). Most students were single (62; 58%). A large proportion of the students identified as religious (61; 57%), with only four identifying as Orthodox (4; 4%).

The first aim of this study was to evaluate nursing students’ clinical learning quality regarding tutorial strategies quality, learning opportunities, self-directed learning, safety and nursing care quality, and learning environment quality before and after their mental health nursing practice. To evaluate nursing students’ perceptions of all this, a one-way repeated measures analysis of variance was utilized.

The findings show a difference between nursing students’ perceptions of clinical learning quality before and after mental health practical learning (F (5,102) = 9.26, *p* = 0.00, ηp2 = 0.31). To further examine this and determine which aspects demonstrate a difference, see Table 2.

Table 2 shows that overall, the means of the clinical learning quality in mental nursing experience was medium-to-high. A significant effect was found when comparing two points in time regarding tutorial strategies quality (F (1′66) = 16.71, *p* = 0.000, ηp2 = 0.20). The students’ mean of tutorial strategies quality was lower after experiencing work in mental health wards than before doing so. There was no difference in the other aspects of clinical learning quality in the mental nursing experience (self-directed learning, safety and nursing care quality, and learning environment quality).

The second aim of this study was to establish if the grade of a theoretical course in mental health nursing and the student’s perception of their theoretical knowledge level predict the grade of mental health nursing practice. For that, a multiple regression analysis was conducted. The variables examined were the grade of a theoretical course in mental health nursing and the student’s perception of their theoretical knowledge level in mental health nursing before the mental health nursing experience. As Table 3 shows, the regression analysis explains 23% of the variance based on the variables (F (2,93) = 14.18, *p* < 0.00).

Table 3 shows that only the grade of a theoretical course in mental health nursing predicted the grade of a mental health nursing practice. Perception of participants’ theoretical knowledge level in mental health nursing did not predict this grade.

We conducted a Pearson correlation analysis to detect relations among clinical learning quality regarding tutorial strategies quality, learning opportunities, and self-directed learning, and whether they mediate learning environment quality and safety and nursing care quality after mental health nursing practice. For the results, see Table 4.

Table 4 shows that significant positive relationships were found between clinical learning quality in these aspects.

**Figure 1:**
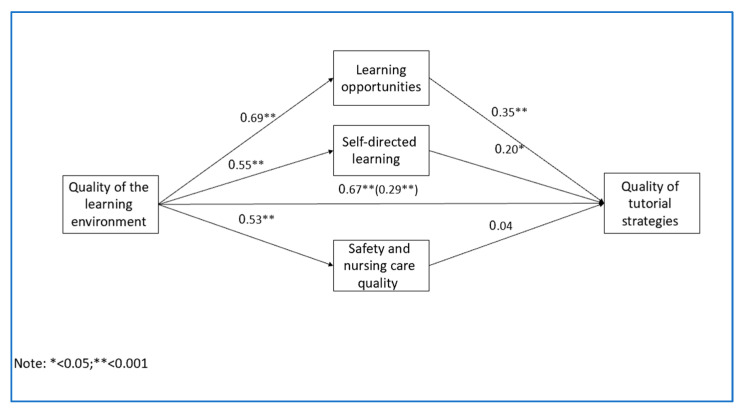
Parallel mediation model.

To explore the third research aim, a model where learning opportunities, self-directed learning and safety, and nursing care quality mediate learning environment quality and tutorial strategies quality following mental health nursing practice, we conducted Model 4 of Process macro [28] mediation analysis. For the research model, see Figure 1.

Figure 1 shows that learning environment quality significantly predicted learning opportunities, self-directed learning, safety and nursing care quality, and tutorial strategies quality. Moreover, learning opportunities and self-directed learning predicted the quality of tutorial strategies. However, safety and nursing care quality did not significantly predict tutorial strategies quality. To further explore the mediation analyses of the research model, see Table 5.

Table 5 indicates that quality of the learning environment is positively related to increased learning opportunities, which in turn is positively related to tutorial strategies quality. The significance test of the indirect effect using a confidence interval yielded a significant mediation effect. This finding is equivalent to a statistically significant effect. Thus, learning opportunities mediate the relationship between learning environment quality and tutorial strategies quality.

Also, quality of the learning environment is positively related to self-directed learning, which in turn was positively significantly related to tutorial strategies quality. The indirect effect was not significant. Thus, self-directed learning does not mediate the relationship between learning environment quality and tutorial strategies quality. In addition, learning environment quality is positively related to safety and nursing care quality, which was not significantly related to tutorial strategies quality, and the indirect effect was insignificant. Thus, safety and nursing care quality do not mediate the relationship between learning environment quality and tutorial strategies quality.

## 4. Discussion

The research aims were to assess the clinical learning quality for nursing students across various aspects, both before and after mental health nursing practice, investigate the predictive value of theoretical course grades and student perceptions on the grade of a mental health nursing practice, and explore how learning opportunities, self-directed learning, and safety and nursing care quality act as mediators between learning environment and tutorial strategies quality following mental health nursing practice.

First, the study found a significant effect when comparing two points in time of students’ perspectives of clinical learning quality in mental nursing experience regarding the aspects of tutorial strategies quality. The students’ mean tutorial strategies quality report was lower after conducting a mental health nursing practice course than before. Dissimilar to our findings, a pre/post-test survey administered to Australian pre-registration nursing students revealed significant improvements in mental health therapeutic engagement, mental health assessment skills, and mental health placement preparedness [29]. It is important to address this issue since clinical learning quality is a key influence on nursing students’ emotional well-being [3]. There is also a need for effective tutorial strategies since it is essential to enhance critical thinking and the accuracy of diagnostic reasoning among nursing students, and intensive clinical tutorials were found to be effective in improving diagnostic reasoning accuracy among nursing students [30]. There is a need for greater consideration of nursing students’ understanding of mental health since the research review found that nursing students’ knowledge of mental health was impacted by their experience of mental health content, and mental health nursing ranked among the lowest specialties in social prestige, student interest, and preferred area of work upon graduation [31]. Mental health settings can provide valuable and quality clinical learning environments for nursing students [9]. However, the decline in the quality of tutorial strategies after mental health practical learning underscores the necessity for dynamic teaching methodologies in clinical learning quality. It is important to continually adapt and enhance instructional approaches to align with the evolving demands of mental health nursing practice.

Second, the study found that students’ grades in a theoretical mental health nursing course were predictive of the grades attained in a mental health nursing practice. In a study conducted at a nursing university in the Philippines, similar results indicated that success in theoretical classroom instruction positively impacted the clinical or practical phase of nursing education [21]. Another study investigated the obstacles delaying the application of theoretical knowledge in clinical contexts, highlighting insufficient theoretical understanding as a significant barrier to effective performance in practical settings [32]. Another study’s results provide preliminary evidence that the concept mapping approach can be useful to help mental health nursing students visualize their learning progress and encourage the integration of theoretical knowledge with clinical knowledge [33]. Research has indicated that the perceived discrepancy between real-world clinical experiences and the ideal theoretical practices taught at universities exacerbates the theory–practice gap [34].

Third, the study found that learning opportunities serve a mediating role in the quality of learning environments and tutorial strategies, which holds critical implications for nursing education and practice. Various perspectives in the literature have explored the connection between learning environment quality and tutorial strategies. In line with constructivist learning theory, the significance of learning opportunities has been emphasized, indicating that teaching quality indirectly influences student achievement [35]. Additionally, the literature emphasizes the value of learning opportunities in various clinical areas for fostering clinical knowledge and higher-order thinking skills among nursing students [36]. Effective clinical teaching approaches, such as active learning strategies and intensive clinical tutorials, have been emphasized as essential for developing nursing students’ competencies and core clinical skills [37]. Active learning tutorial strategies are student-centered approaches that require students to participate and cooperate in the teaching and learning process [38]. Employing such learning strategies not only facilitates a seamless transition into the workforce but also accommodates diverse learning styles [39]. Moreover, it is worthwhile acknowledging the significance of mental health settings as valuable clinical learning environments for nursing students [9]. The current study stands out as it addresses a significant gap in the existing literature, exploring in-depth variables such as learning opportunities, self-directed learning, and safety and nursing care quality act as mediators between learning environment quality and tutorial strategies in the context of mental health nursing practice.

## 5. Conclusions

This study contributes insights into clinical learning quality and its impact on nursing education in the context of mental health nursing practice. Firstly, the study demonstrated a decrease in the mean quality of tutorial strategies post-practical learning, highlighting the need for a motivated nature of education methodologies in clinical learning. This may emphasize the need for continuous adaptation and enhancement of instructional approaches to meet the evolving demands of mental health nursing practice. Additionally, the exploration of how learning opportunities, self-directed learning, safety, and nursing care quality function as mediators between learning environment quality and tutorial strategies quality has uncovered advanced insights with significant implications for nursing education and practice. The identified mediating role of learning opportunities highlights the crucial influence of a supportive and enriching learning environment in fostering effective tutorial strategies in mental health practice.

## 6. Limitations and Recommendations for Future Research

The study has several limitations: Its reliance on a relatively small sample of undergraduate nursing students from a single Israeli institution may limit the generalizability of the findings to a broader population. Future research should include a larger, more diverse participant sample to ensure the representativeness of the results. Another limitation relates to the focus on mental health practice within nursing education, as the study’s findings may not fully reflect the experiences and challenges encountered in other specialty clinical nursing areas, such as which aspects of tutoring quality are being used and assessed. Incorporating a broader range of such areas could provide a more comprehensive understanding of the issue. An additional limitation is the study’s reliance on self-reported questionnaires to evaluate perceptions of learning quality, for the possibility of response bias and subjectivity. Future research could incorporate objective measures or observational methods to complement self-reported data, or use a complex mixed-method design [40] providing a more holistic understanding of clinical learning quality.

## Figures and Tables

**Table 1:** Frequency and percentage of background characteristics.

Background Characteristics		*N* = 107	
		*n*	%
Gender	Male	20	19
	Female	87	81
Status	Single	62	58
	Married	40	37
	Divorced	5	5
Religiosity	Secular	14	13
	Traditional	28	26
	Religious	61	57
	Orthodox	4	4
	Mean (SD)	Minimum	Maximum
Perceived level of knowledge in mental health nursing after the theoretical course learning	3.88 (0.7)	2	5
Scores of the mental health nursing practice	92 (5.63)	70	100

**Table 2:** Means and standard deviations for nursing students’ mental health practical learning perspectives at two points: before (T1) and after (T2).

Variables		Pre-Mental Health Nursing Practice Mean (SD)	Post-Mental Health Nursing Practice Mean (SD)	*p*
Clinical Learning Quality Evaluation	Tutorial strategies quality	2.79 (0.24)	2.59 (0.53)	**
	Learning opportunities	2.69 (0.35)	2.61 (0.46)	--
	Self-directed learning	2.38 (0.67)	2.47 (0.75)	--
	Safety and nursing care quality	2.67 (0.42)	2.66 (0.39)	--
	Learning environment quality	2.24 (0.56)	2.33 (0.72)	--

Abbreviations: M—mean; SD—standard deviation; * *p* < 0.05; ** *p* < 0.01.

**Table 3:** Multiple regression is used to predict the grade of a mental health nursing practice.

Predictors	B	β	t	R^2^
				0.23
Grade of a theoretical course in mental health nursing	0.33	0.42	4.50 **	
Perception of participants’ theoretical knowledge level in mental health nursing	1.37	0.16	1.74	

** *p* < 0.00.

**Table 4:** Relations among clinical learning quality components.

Variables	Tutorial Strategies Quality	Learning Opportunities	Self-Directed Learning	Safety and Nursing Care Quality	Learning Environment Quality
Tutorial strategies quality	1	0.72 **	0.62 **	0.56 **	0.67 **
Learning opportunities		1	0.66 **	0.70 **	0.69 **
Self-directed learning			1	0.52 **	0.55 **
Safety and nursing care quality				1	0.53 **
Learning environment quality					1

Abbreviations: * *p* < 0.05; ** *p* < 0.01.

**Table 5:** Parallel mediation model, direct and indirect effect.

Variables	Beta	B	SE	t	*p*	95%CI
Predicting the mediator learning opportunities						
Learning environment quality	0.69	0.44	0.04	9.82	**	[0.35, 0.53]
Predicting the mediator self-directed learning						
Learning environment quality	0.55	0.58	0.08	6.88	**	[0.41, 0.75]
Predicting the mediator safety and nursing care quality						
Learning environment quality	0.53	0.29	0.04	6.50	**	[0.20, 0.38]
Predicting the outcome, tutorial strategies quality						
Learning environment quality	0.29	0.21	0.06	3.33	**	[0.08, 0.34]
Learning opportunities	0.35	0.41	0.12	3.26	**	[0.16, 0.66]
Self-directed learning	0.20	0.14	0.05	2.42	**	[0.02, 0.26]
Safety and nursing care quality	0.04	0.06	0.11	0.22	-	[−0.17, 0.30]
Indirect effect via						
Learning opportunities	0.24	0.18	0.09	-	*	[0.05, 0.43]
Self-directed learning	0.11	0.08	0.08	-	-	[−0.01, 0.30]
Safety and nursing care quality	0.02	0.07	0.07	-	-	[−0.11, 0.16]

Notes: * *p* < 0.05; ** *p* < 0.00.

## Data Availability

The data of this article cannot be shared publicly due to the privacy concerns of the people who participated in the study.

## Public Involvement Statement

No public involvement in any aspect of this research.

## Guidelines and Standards Statement

The Transparent Reporting of The study’s reporting framework was guided by the checklist provided in the Evaluations with Nonrandomized Designs (TREND) statement from Equator.
